# Combined assessment of ASPECTS and collateral status predict outcomes after endovascular therapy in patients with large ischemic stroke

**DOI:** 10.3389/fneur.2026.1887827

**Published:** 2026-09-10

**Authors:** Wei Deng, Zhengqiao Bao, Jun Han, Yanli Sun, Peiyang Zhou, Guangxian Nan

**Affiliations:** 1 Department of Neurology, China-Japan Union Hospital of Jilin University, Changchun, Jilin, China; 2 Department of Neurology, Xiangyang No. 1 People’s Hospital, Hubei University of Medicine, Xiangyang, Hubei, China; 3 Department of General Practice, Xiangyang No. 1 People’s Hospital, Hubei University of Medicine, Xiangyang, Hubei, China; 4 Department of Endocrinology, The First Affiliated Hospital of Anhui Medical University, Hefei, Anhui, China

**Keywords:** ASPECTS, collateral status, endovascular therapy, large ischemic stroke, outcomes

## Abstract

**Background:**

Both the Alberta Stroke Program Early Computed Tomography Score (ASPECTS) and collateral status are well-established predictors of clinical outcomes in patients with large ischemic stroke undergoing endovascular therapy (EVT); however, they have traditionally been assessed independently. This study aimed to evaluate the prognostic value of a combined imaging score, the AS-TCIS score, which integrates ASPECTS and collateral status into a unified framework.

**Methods:**

This study is a subanalysis of a prospective multicenter cohort including patients with ASPECTS ≤5 enrolled between November 2021 and February 2023. The AS-TCIS score was constructed by integrating the 5-point ASPECTS with collateral status, including the 3-point Tan score and the 8-point comprehensive venous outflow (CVO) score, yielding a 16-point composite scale. The primary outcomes were 90-day functional outcome (modified Rankin Scale [mRS] 0–3) and mortality.

**Results:**

Of 750 patients, 287 were eligible (median age 67 years; 58.5% male; median AS-TCIS score 8). In multivariable models, each 1-point increase in AS-TCIS was associated with higher odds of mRS 0–3 (OR 1.38; 95% CI 1.24–1.56) and mRS 0–2 (OR 1.30; 95% CI 1.17–1.47), and lower odds of mortality (OR 0.74; 95% CI 0.66–0.82), with no significant association with symptomatic intracranial hemorrhage (OR 0.92; 95% CI 0.82–1.03). AS-TCIS also demonstrated a linear association with functional outcome and mortality. Compared with ASPECTS, Tan score, and their combination, AS-TCIS showed consistently higher AUCs and improved model fit across all outcomes.

**Conclusion:**

The AS-TCIS score, a composite of ASPECTS and collateral status, was independently associated with 90-day outcomes in patients with large ischemic stroke undergoing EVT. It may provide incremental value for risk stratification and outcome prediction in acute stroke imaging assessment.

## Introduction

Advanced perfusion imaging, such as computed tomography perfusion (CTP), has greatly facilitated the development of endovascular therapy (EVT) for acute ischemic stroke (AIS) (1). It has long been regarded as the standard modality for treatment selection, with patients demonstrating a larger mismatch between infarct core and penumbra considered more suitable candidates for EVT (1, 2). Recently, several randomized controlled trials (RCTs) have confirmed the superiority of EVT over standard medical treatment (SMT) in patients with large ischemic stroke (3–7). However, it is important to recognize that rates of functional independence remain low (14–30%) (8), including in trials where patient selection was based solely on an Alberta Stroke Program Early Computed Tomography Score (ASPECTS) below 6 (5–7). Moreover, noncontrast CT (NCCT) and CT angiography (CTA) are widely used as the primary imaging modalities in patients with large ischemic stroke, particularly when CTP is unavailable. Evidence also suggests that the use of CTP compared with NCCT/CTA may have little or no effect on clinical outcomes after EVT (9, 10). Consequently, optimal patient selection for EVT remains a major challenge in real-world clinical practice.

Although ASPECTS is widely used to assess early ischemic changes, it provides limited information regarding the extent of viable penumbra and collateral circulation. Previous studies have demonstrated a close relationship between ASPECTS and collateral status with CT perfusion (CTP)-derived ischemic core volume (11, 12). To simplify therapeutic imaging assessment, the ASCO score, which combines ASPECTS and arterial collateral status based on single-phase CTA (sCTA), has been shown to outperform CTP-derived ischemic core volume models in predicting good functional outcomes in patients with low ASPECTS (13). Furthermore, Koji Tanaka et al. performed a *post hoc* analysis of the AcT trial and demonstrated that the mCTA-ACE score, integrating ASPECTS with multiphase CTA (mCTA)-based arterial collateral assessment, can effectively predict outcomes after EVT, with performance comparable to ischemic core volume (14). However, these approaches do not account for venous collateral status, which has been shown to be independently associated with functional independence (15–17). In addition, several studies have demonstrated that comprehensive venous outflow (CVO) is strongly associated with successful reperfusion and favorable clinical outcomes in patients with acute ischemic stroke undergoing EVT (18, 19). Despite these findings, the prognostic value of a combined imaging score integrating ASPECTS and both arterial and venous collateral status (AS-TCIS score) remains unclear in patients with large ischemic stroke.

In this study, we performed a subanalysis of a prospective multicenter cohort to evaluate the prognostic value of the AS-TCIS score derived from single-phase CTA (sCTA) in patients with large ischemic stroke undergoing EVT. We hypothesized that higher AS-TCIS scores are associated with better functional outcomes after EVT.

## Methods

### Study population

This study was a subanalysis of a nationwide, prospective cohort study involving 38 comprehensive stroke centers in China, which evaluated the effectiveness and safety of EVT versus SMT in patients with large ischemic stroke between November 1, 2021, and February 8, 2023 (20). The study was approved by the ethics committees of all participating centers, and informed consent was obtained from all patients or their legally authorized representatives. The study was conducted in accordance with the Declaration of Helsinki. The main inclusion criteria were:age ≥ 18 years, AIS patients due to anterior circulation large vessel occlusion and undergoing EVT, large ischemic stroke on NCCT (ASPECTS of 0–5), completion of baseline head CTA at admission, within 24 h of stroke onset or last known within 24 h. The main exclusion criteria were premorbid modified Rankin Scale (mRS) > 2, absence of 90-day follow-up or key baseline data, and the presence of serious, advanced, or terminal illness unrelated to AIS.

### Variables and imaging analysis

We collected data on baseline characteristics, stroke risk factors, imaging features, time metrics, and clinical outcomes. Comparative analyses were performed according to the median AS-TCIS score. Imaging data were assessed at an independent core imaging laboratory. Two experienced investigators, blinded to clinical outcomes, independently reviewed all CTA images, and any discrepancies were resolved by a third senior investigator.

Cerebral collateral status was evaluated using both the Tan score and the CVO score, which incorporates assessment of both cortical (COVES) and deep (ICV) venous drainage. The Tan score ranges from 0 (no collateral supply to the occluded middle cerebral artery territory) to 3 (complete collateral supply). The CVO score ranges from 0 (no opacification of one deep and three cortical venous pathways) to 8 (full opacification of these venous drainage pathways) (21). The 5-point ASPECTS was combined with collateral assessment, including the 3-point Tan score and the 8-point CVO score, to yield a composite 16-point AS-TCIS score.

### Outcomes measurements

The primary outcomes were favorable functional outcome (mRS 0–3) and mortality at 90 days. Secondary outcomes included functional independence (mRS 0–2) and symptomatic intracranial hemorrhage (sICH), as defined by the Heidelberg Bleeding Classification. Successful reperfusion was defined as Thrombolysis in Cerebral Infarction (TICI) grade 2b–3.

### Statistical analysis

Statistical analyses were performed using R software version 4.4.2 (R Foundation for Statistical Computing) and SPSS version 27.0 for Windows. All statistical tests were two-sided, and a *p* value <0.05 was considered statistically significant. Missing baseline data were handled using multiple imputation, as described in the Supplementary material. Continuous variables were expressed as mean ± standard deviation or median (interquartile range), as appropriate, and compared using Student’s t-test or the Wilcoxon rank-sum test. Categorical variables were summarized as counts (percentages) and compared using the χ^2^ test or Fisher’s exact test, as appropriate.

The AS-TCIS score was analyzed as a continuous variable in the primary analyses, with additional exploratory analyses performed using a median-based dichotomization (≤8 vs. >8). Associations between the AS-TCIS score and binary outcomes (mRS 0–3, mRS 0–2, sICH, and mortality) were assessed using logistic regression models. Three sequential models were constructed: Model 1 was unadjusted; Model 2 was adjusted for age and sex; and Model 3 further adjusted for prior stroke, diabetes, hypertension, glucose, baseline NIHSS, onset to recanalization time, intravenous thrombolysis, occlusion site, and successful reperfusion. Odds ratios (ORs) and 95% confidence intervals (CIs) were reported. Restricted cubic spline models with four knots were used within Model 3 to explore potential non-linear associations between the AS-TCIS score and outcomes. Predictive performance was evaluated using the area under the receiver operating characteristic curve (AUC), while model fit was assessed using the Akaike Information Criterion (AIC) and Bayesian Information Criterion (BIC). Finally, incremental predictive value was quantified using the Net Reclassification Index (NRI) and Integrated Discrimination Improvement (IDI).

## Results

### Baseline characteristics

A total of 750 patients from 38 comprehensive stroke centers in China were screened, of whom 287 patients (168 men, 58.5%) undergoing EVT were included in the analysis (Supplementary Figure S1, study flowchart). The median age was 67 years (IQR, 58–77), and the median baseline NIHSS score was 17 (IQR, 14–21). Compared with patients with AS-TCIS ≤8, those with AS-TCIS >8 had lower median glucose levels (7.1 vs. 7.6 mmol/L), lower baseline NIHSS (15 vs. 18), a higher proportion of distal occlusions (M1: 62.2% vs. 34.2%; M2: 18.5% vs. 6.6%), and a greater proportion of favorable collateral status (ASITN/SIR grade 3: 25.2% vs. 13.2%) (Table 1). Other baseline characteristics were well balanced between the two groups.

**Table 1:** Characteristics of patients.

Variables	Overall (*n* = 287)	AS-TCIS score ≤8 (*n* = 152)	AS-TCIS score >8 (*n* = 135)	*p* value
Age, y, median (IQR)	67 (58–77)	67 (58–75)	67 (57–77)	0.701
Sex, male, *n* (%)	168 (58.5)	90 (59.2)	78 (57.8)	0.9
History, *n* (%)				
Prior stroke	39 (13.6)	23 (15.1)	16 (11.9)	0.524
Smoking	94 (32.8)	50 (32.9)	44 (32.6)	1
Hypertension	169 (58.9)	88 (57.9)	81 (60.0)	0.809
Hyperlipidemia	61 (21.3)	28 (18.4)	33 (24.4)	0.271
Diabetes	46 (16.0)	27 (17.8)	19 (14.1)	0.491
Atrial fibrillation	112 (39.0)	59 (38.8)	53 (39.3)	1
SBP, mmHg, median (IQR)	145 (125–160)	145 (127–160)	144 (125–160)	0.455
DBP, mmHg, median (IQR)	85 (75–95)	84 (75–95)	86 (75–95)	0.895
Glucose, mmol/l, median (IQR)	7.3 (6.1–9.1)	7.6 (6.5–9.5)	7.1 (5.9–8.6)	0.012
Baseline NIHSS, median (IQR)	17 (14–21)	18 (15–22)	15 (13–19)	<0.001
TOAST etiology, *n* (%)				0.952
LAA	85 (29.6)	45 (29.6)	40 (29.6)	
CE	153 (53.3)	82 (53.9)	71 (52.6)	
Others	49 (17.1)	25 (16.4)	24 (17.8)	
Occlusion site, *n* (%)				<0.001
ICA	116 (40.4)	90 (59.2)	26 (19.3)	
M1	136 (47.4)	52 (34.2)	84 (62.2)	
M2	35 (12.2)	10 (6.6)	25 (18.5)	
Tandem occlusion, *n* (%)	23 (8.0)	13 (8.6)	10 (7.4)	0.89
Anesthesia, local, *n* (%)	247 (86.1)	127 (83.6)	120 (88.9)	0.258
Time metrics, min, median (IQR)				
Onset to imaging time	342 (183–529)	327 (179–485)	345 (184–567)	0.429
Onset to puncture time	420 (271–620)	397 (261–591)	440 (284–658)	0.115
Onset to recanalization time	503 (356–725)	480 (335–706)	535 (374–758)	0.137
ASITN/SIR, *n* (%)				<0.001
0–1	128 (44.6)	92 (60.5)	36 (26.7)	
2	105 (36.6)	40 (26.3)	65 (48.1)	
3	54 (18.8)	20 (13.2)	34 (25.2)	
IVT, *n* (%)	75 (26.1)	40 (26.3)	35 (25.9)	1
Successful reperfusion, *n* (%)	244 (85.0)	126 (82.9)	118 (87.4)	0.366

SBP, systolic blood pressure; DBP, diastolic blood pressure; NIHSS, National Institutes of Health Stroke Scale; LAA, large-artery atherosclerosis; ICA, Internal carotid artery; TOAST, Trial of Org10172 in Acute Stroke Treatment; ASITN/SIR, grade indicates the American Society of Interventional and Therapeutic Neuroradiology/Society of Interventional Radiology collateral score; IVT, intravenous thrombolysis; successful reperfusion defined as TICI 2b–3; IQR, interquartile range.

### Associations between AS-TCIS score and outcomes

An elevated AS-TCIS score was consistently associated with better clinical outcomes after EVT across all three models. In Model 1, each 1-point increase in the AS-TCIS score was associated with higher odds of mRS 0–3 (OR 1.34; 95% CI 1.23–1.47; *p* < 0.001) and mRS 0–2 (OR 1.31; 95% CI 1.18–1.45; *p* < 0.001). These associations remained significant after adjustment for age and sex in Model 2, and further persisted in Model 3 after additional adjustment for clinical covariates (OR 1.38 and 1.30, respectively; both *p* < 0.001). A significant reduction in mortality was also observed with increasing AS-TCIS scores (Model 1: OR 0.75; 95% CI 0.69–0.82; *p* < 0.001; Model 3: OR 0.74; 95% CI 0.66–0.82; *p* < 0.001). Consistent results were observed when the AS-TCIS score was analyzed as a categorical variable. However, no significant association was found between the AS-TCIS score and sICH in Model 3 (OR 0.92; 95% CI 0.82–1.03; *p* = 0.170) (Table 2).

**Table 2:** Association between AS-TCIS score and outcomes.

Variables	Model 1		Model 2		Model 3	
	OR (95% CI)	*p* value	OR (95% CI)	*p* value	OR (95% CI)	*p* value
mRS 0–3						
Continuous	1.34 (1.23–1.47)	<0.001	1.37 (1.25–1.51)	<0.001	1.38 (1.24–1.56)	<0.001
Categorical						
AS-TCIS ≤8	–		–		–	
AS-TCIS >8	5.71 (3.38–9.84)	<0.001	7.06 (4.01–12.83)	<0.001	6.47 (3.33–13.10)	<0.001
mRS 0–2						
Continuous	1.31 (1.18–1.45)	<0.001	1.30 (1.18–1.45)	<0.001	1.30 (1.17–1.47)	<0.001
Categorical						
AS-TCIS ≤8	–		–		–	
AS-TCIS >8	4.39 (2.39–8.45)	<0.001	4.53 (2.45–8.76)	<0.001	4.20 (2.04–9.07)	<0.001
sICH						
Continuous	0.90 (0.82–0.99)	0.038	0.91 (0.82–1.00)	0.044	0.92 (0.82–1.03)	0.170
Categorical						
AS-TCIS ≤8	–		–		–	
AS-TCIS >8	0.36 (0.17–0.73)	0.007	0.35 (0.16–0.72)	0.006	0.40 (0.17–0.91)	0.033
Mortality						
Continuous	0.75 (0.69–0.82)	<0.001	0.74 (0.67–0.80)	<0.001	0.74 (0.66–0.82)	<0.001
Categorical						
AS-TCIS ≤8	–		–		–	
AS-TCIS >8	0.17 (0.10–0.29)	<0.001	0.14 (0.08–0.24)	<0.001	0.16 (0.08–0.31)	<0.001

Model 1: no adjustment for covariates. Model 2: adjusted for age and sex. Model 3: adjusted for age, sex, prior stroke, diabetes, hypertension, glucose, baseline NIHSS, onset to recanalization time, intravenous thrombolysis, occlusion site, and successful reperfusion.

**Figure 1:**
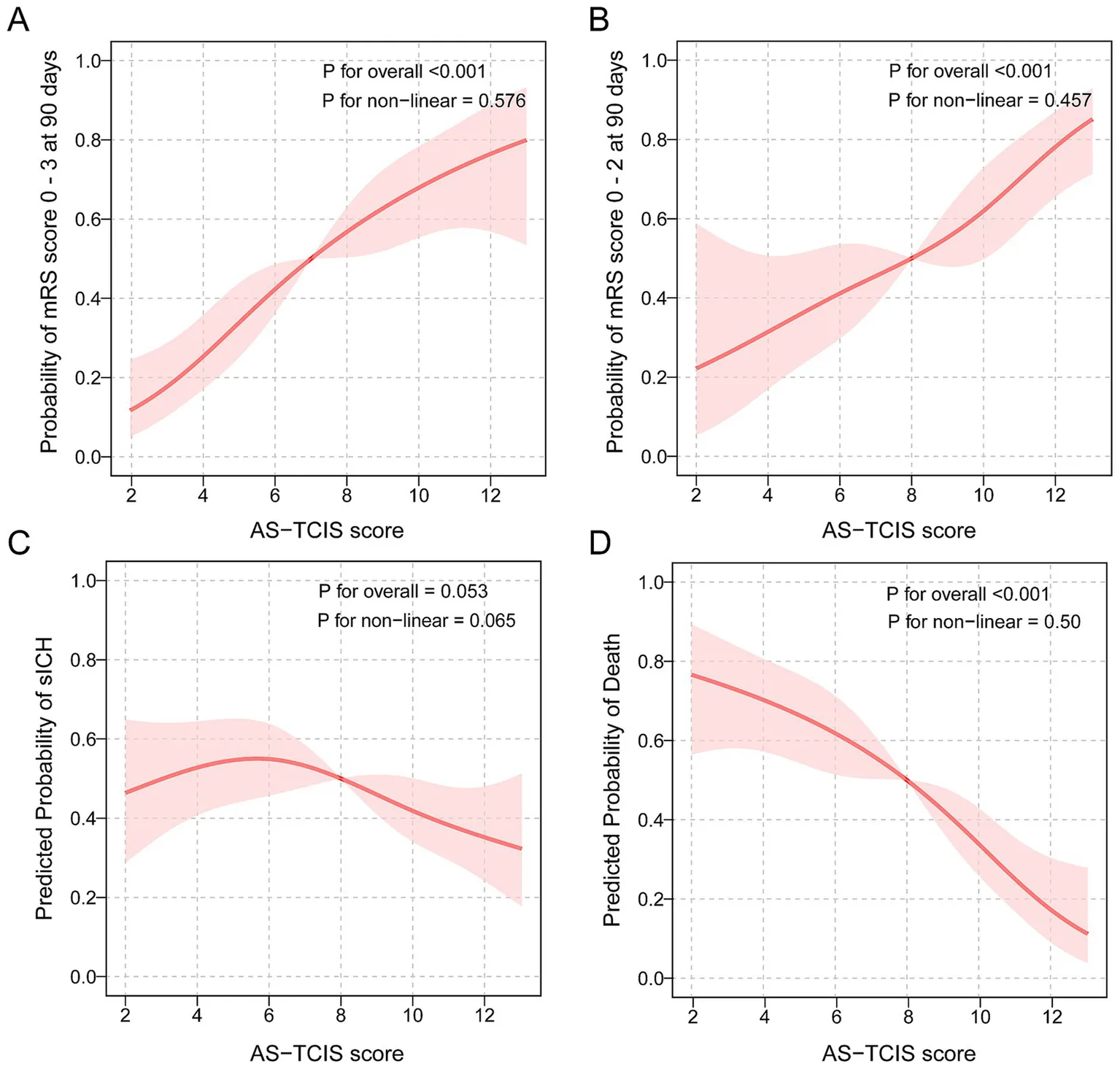
Changes in predicted probability of outcomes according to continuous AS-TCIS score. Associations between **(A)** TCIS score and modified Rankin Scale (mRS) score 0–3, **(B)** mRS score 0–2, **(C)** symptomatic intracranial hemorrhage (sICH), and **(D)** mortality.

Restricted cubic spline analysis demonstrated a significant positive linear association between the AS-TCIS score and the probability of mRS 0–3 and mRS 0–2 at 90 days, with no evidence of non-linearity (*p* < 0.001; Figures 1A,B). In contrast, the AS-TCIS score was significantly negatively associated with 90-day mortality (p < 0.001) and showed no significant association with sICH (*p* = 0.053; Figures 1C,D).

### Predictive performance of AS-TCIS score

**Figure 2:**
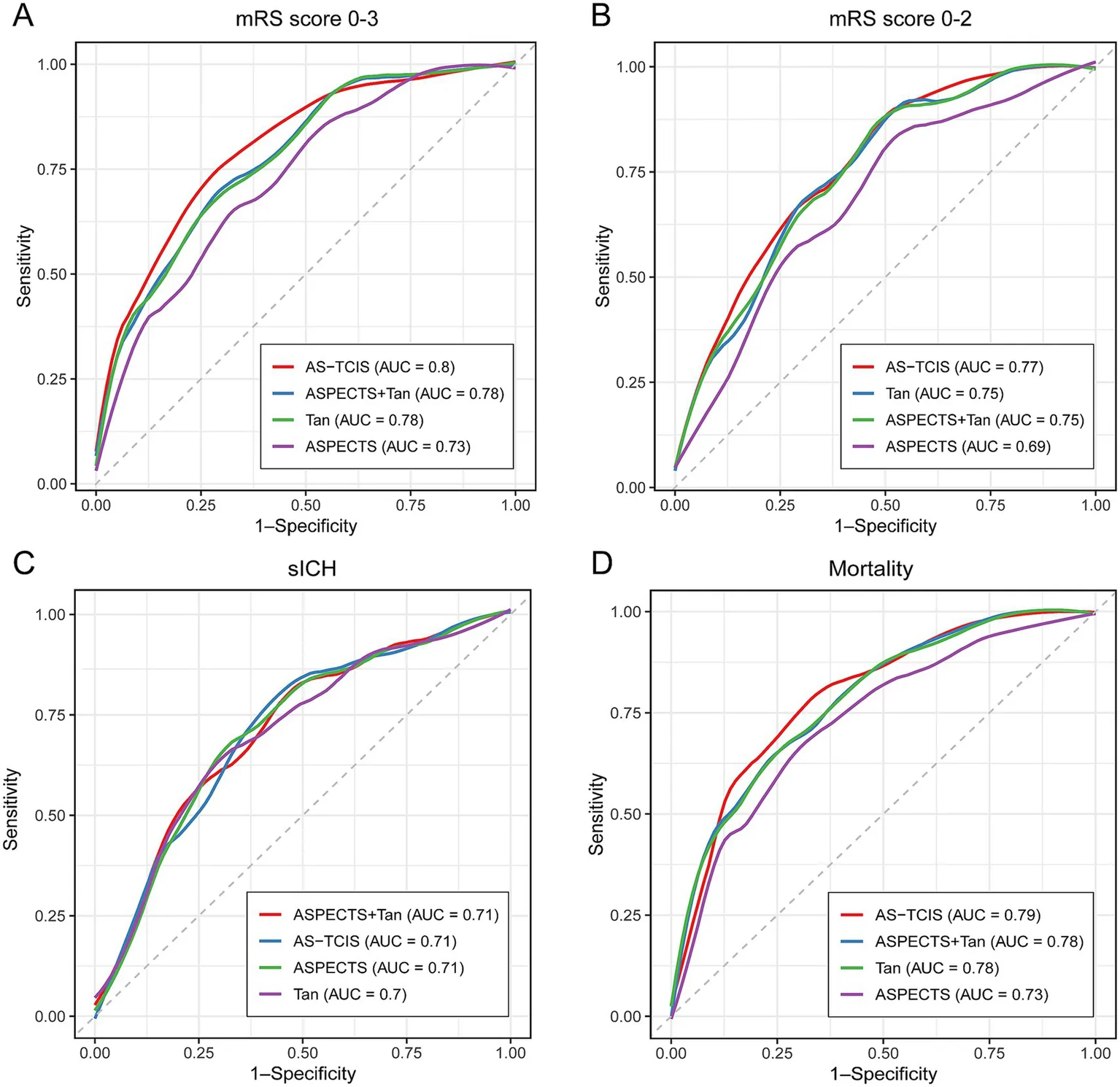
Comparison of predictive accuracy (ROC curves) of AS-TCIS score and other imaging predictors for outcome. Receiver operation characteristic curves of Alberta Stroke Program Early Computed Tomography Score (ASPECTS), Tan Collateral Score (Tan), the combination of ASPECTS and Tan, the AS-TCIS score (ASPECTS+Tan+CVO) for **(A)** modified Rankin Scale (mRS) score 0–3, **(B)** mRS score 0–2, **(C)** symptomatic intracranial hemorrhage (sICH), and **(D)** mortality.

ROC curve analysis demonstrated that the AS-TCIS score showed the highest overall discriminative performance for clinical outcomes, with AUCs of 0.80 for mRS 0–3, 0.77 for mRS 0–2, 0.79 for mortality, and 0.71 for sICH (Figure 2). In addition, the AS-TCIS score yielded improved model fit, as indicated by lower AIC and BIC values compared with ASPECTS, Tan score, or their combination (Supplementary Table S1 in Supplementary Data). DeLong test results showed that the AS-TCIS score had significantly higher AUCs for mRS 0–3 (vs. ASPECTS *p* = 0.015; vs. Tan *p* = 0.048; vs. ASPECTS+Tan *p* = 0.287), mRS 0–2 (*p* = 0.023; *p* = 0.062; *p* = 0.350), and mortality (*p* = 0.011; *p* = 0.038; *p* = 0.279), whereas no significant differences were observed for sICH (*p* = 0.711; *p* = 0.853; *p* = 0.936).

### Model improvement with AS-TCIS score

The addition of the AS-TCIS score to the basic model significantly improved discrimination for mRS 0–3, with the C statistic increasing from 0.783 to 0.847 (*p* = 0.001). However, no significant improvement was observed in continuous NRI (0.806, *p* = 0.500) or IDI (0.118, *p* = 0.488). Similar findings were observed for mRS 0–2, where the C statistic increased from 0.728 to 0.806 (*p* = 0.003), while NRI (0.664, *p* = 0.510) and IDI (0.087, *p* = 0.490) remained non-significant. For mortality, the addition of AS-TCIS significantly improved the C statistic (0.778 vs. 0.841; *p* < 0.001), whereas NRI (0.677, *p* = 0.484) and IDI (0.100, *p* = 0.498) showed no significant changes. In contrast, no significant improvement was observed for sICH across any metric (Table 3).

**Table 3:** Model improvement with AS-TCIS score.

Model	C statistic Estimate (95% CI)	*P* value	NRI (continuous) Estimate (95% CI)	*p* value	IDI Estimate (95% CI)	*p* value
mRS 0–3						
Basic model	0.783 (0.73–0.835)		Reference		Reference	
Basic model + AS-TCIS	0.847 (0.803–0.89)	0.001	0.806 (−0.247–0.236)	0.500	0.118 (−0.039–0.036)	0.488
mRS 0–2						
Basic model	0.728 (0.665–0.791)		Reference		Reference	
Basic model + AS-TCIS	0.806 (0.748–0.863)	0.003	0.664 (−0.274–0.257)	0.510	0.087 (−0.032–0.038)	0.490
sICH						
Basic model	0.705 (0.618–0.792)		Reference		Reference	
Basic model + AS-TCIS	0.718 (0.636–0.799)	0.466	0.244 (−0.293–0.321)	0.492	0.004 (−0.01–0.009)	0.536
Mortality						
Basic model	0.778 (0.723–0.833)		Reference		Reference	
Basic model + AS-TCIS	0.841 (0.796–0.886)	<0.001	0.677 (−0.227–0.206)	0.484	0.1 (−0.037–0.031)	0.498

NRI, Net Reclassification Improvement; IDI, Integrated Discrimination Improvement; Basic model: age, sex, prior stroke, diabetes, hypertension, glucose, baseline NIHSS, onset to recanalization time, intravenous thrombolysis, occlusion site, and successful reperfusion.

## Discussion

This study demonstrates that the AS-TCIS score, a novel imaging composite integrating ASPECTS and collateral status based on single-phase CTA, is significantly associated with clinical outcomes in patients with large ischemic stroke undergoing EVT. To our knowledge, this is the first study to systematically integrate both arterial and venous collateral information with ASPECTS within a unified scoring framework. Our findings provide evidence that the AS-TCIS score is a simple and clinically applicable imaging tool that may improve prognostic stratification in routine clinical practice.

Previous studies have demonstrated the prognostic value of combining ASPECTS with arterial collateral assessment (Tan score or mCTA-ACE score) in acute ischemic stroke, with predictive performance comparable to that of CTP-derived ischemic core volume (12, 22). However, cerebral collateral circulation, which is jointly determined by arterial inflow and venous outflow, plays a critical role in maintaining perfusion to the ischemic penumbra, limiting infarct core expansion, and enhancing the therapeutic benefit of EVT (23, 24). Emerging evidence further suggests that venous outflow is independently associated with functional outcomes, even after accounting for arterial collateral status (13, 14). In the present study, the AS-TCIS score integrates both arterial and venous collateral information derived from sCTA in patients with large ischemic stroke, thereby providing a more comprehensive representation of global perfusion status (25). In parallel, ASPECTS reflects early ischemic tissue changes on non-contrast CT, primarily driven by cytotoxic edema and early net water uptake in hypoperfused brain regions (26). The integration of ASPECTS with collateral assessment may therefore capture complementary aspects of infarct evolution across different temporal and physiological stages, potentially offering a more robust surrogate of salvageable brain tissue.

In our study, patients with AS-TCIS scores >8 exhibited more favorable baseline characteristics compared with those with scores ≤8, including lower glucose levels (7.1 vs. 7.6 mmol/L), lower baseline NIHSS scores (15 vs. 18), a higher proportion of distal vessel occlusions (M1: 62.2% vs. 34.2%; M2: 18.5% vs. 6.6%), and a greater prevalence of robust collateral status (ASITN/SIR grade 3: 25.2% vs. 13.2%). These findings are consistent with previous studies indicating that better collateral status is associated with smaller infarct cores and more preserved neurological function at presentation (27–29). Distal occlusions, particularly involving the M2 segment, are more likely to preserve residual perfusion and are frequently accompanied by more developed collateral circulation, which may partly explain the higher AS-TCIS scores observed in these patients (30). In addition, hyperglycemia has been shown to impair collateral recruitment and is associated with worse outcomes in acute ischemic stroke (27, 31), potentially contributing to lower AS-TCIS scores in patients with elevated glucose levels. Importantly, higher AS-TCIS scores were associated with better functional outcomes, suggesting that this score reflects not only baseline clinical severity but also underlying cerebrovascular compensatory capacity. These findings are in line with previous studies reporting that favorable baseline infarct characteristics and collateral status are key determinants of outcome after EVT (9–17, 14, 32). The association between AS-TCIS and sICH observed in univariate analysis was no longer significant after adjustment, likely reflecting confounding by key clinical and procedural factors (e.g., baseline stroke severity and reperfusion status) and limited statistical power due to the relatively low number of hemorrhagic events. Moreover, restricted cubic spline analysis demonstrated a linear, dose-dependent association between AS-TCIS score and both functional independence and survival.

Additionally, the AS-TCIS score demonstrated good predictive performance, with AUCs of 0.80 for mRS 0–3, 0.77 for mRS 0–2, and 0.79 for mortality, and generally outperformed ASPECTS, Tan score, and their combination. However, a previous study by Koji Tanaka et al. reported that the combination of ASPECTS and arterial collaterals was not superior to ASPECTS alone or CTA-based collateral assessments (12). This discrepancy may be partly explained by the additional incorporation of venous collateral information in the AS-TCIS score, underscoring the potential value of comprehensive collateral evaluation in outcome prediction after EVT (15–17, 21, 33, 34). The DeLong test showed that AS-TCIS provided statistically significant improvement over ASPECTS and Tan score for several outcomes; however, comparisons with the combined ASPECTS + Tan model were not consistently significant. Consistent with these findings, incremental model performance analyses demonstrated improvements in C statistics for several outcomes, while NRI and IDI did not show consistent statistical significance, suggesting a modest and outcome-dependent added predictive value of AS-TCIS when incorporated into existing models.

Our findings have several clinical implications. First, the AS-TCIS score is derived entirely from NCCT/sCTA, making it potentially applicable in centers where advanced perfusion imaging is not available. Second, the incorporation of venous collateral assessment is consistent with emerging evidence suggesting that venous outflow may more accurately reflect downstream perfusion and tissue viability than arterial inflow alone (13, 14). Third, in the context of expanding EVT indications to patients with large ischemic stroke, the AS-TCIS score may help refine patient selection beyond conventional ASPECTS-based thresholds and provide additional prognostic stratification in routine clinical practice.

Several limitations should be acknowledged. First, this analysis represents a sub-study of a prospective multicenter cohort; although extensive adjustment for key confounders was performed, residual confounding inherent to observational study designs cannot be fully excluded. Second, although collateral grading demonstrated high interobserver reliability (*κ* > 0.80), variability in CTA acquisition protocols and scanner platforms across participating centers may still introduce measurement heterogeneity. To minimize this, all imaging data were centrally evaluated by three independent, blinded readers. Third, the AS-TCIS score was derived from single-phase CTA, which may not fully capture dynamic venous outflow; future studies incorporating multiphase CTA or CT perfusion imaging may further improve collateral characterization. Fourth, the equal weighting of ASPECTS, Tan score, and venous collateral components is a simplifying assumption that may not reflect their true relative prognostic contributions and should be considered a limitation of this exploratory framework. Finally, the study population was exclusively Chinese, and external validation in more ethnically and geographically diverse cohorts is warranted to enhance generalizability.

In summary, the AS-TCIS score, a composite of ASPECTS and collateral status, was independently associated with 90-day outcomes in patients with large ischemic stroke undergoing EVT. Its predictive performance was comparable to, and in some outcomes slightly better than, conventional imaging markers. These findings support the potential utility of the AS-TCIS score as a practical tool for outcome prediction, particularly in settings where advanced perfusion imaging is not available.

## Data Availability

The raw data supporting the conclusions of this article will be made available by the authors, without undue reservation.
