# Supplementary material for: Combined assessment of ASPECTS and collateral status predict outcomes after endovascular therapy in patients with large ischemic stroke
Source: Front Neurol. 2026 Sep 10;17:1887827. doi: 10.3389/fneur.2026.1887827 (PMC13600863; doi:10.3389/fneur.2026.1887827)
Supplement: Supplementary file 1 [file Supplementary_File_1.docx]

**Supplementary materials**

***Statistical analysis***

Complete data were available for most baseline variables, and the overall proportion of missing data was low. Missing values were observed for SBP and DBP in 1 of 287 patients each (0.3%), glucose in 7 patients (2.4%), onset-to-puncture time in 1 patient (0.3%), and onset-to-recanalization time in 2 patients (0.7%). Complete follow-up data were available for the 90-day modified Rankin Scale score. Missing data were handled using multiple imputation in SPSS version 27 under the assumption that data were missing completely at random. Imputation was performed using a fully conditional specification approach with an iterative Markov chain Monte Carlo algorithm. Five imputed datasets were generated, with ten iterations for each dataset, and the dataset with the highest reliability coefficient was selected for subsequent analyses.

The participating institutional ethics committees are from the following 38 hospitals: the Ethics Committee of the Second Affiliated Hospital of Army Medical University, Bazhong Central Hospital, Bazhong City Traditional Chinese Medicine Hospital, Chongzhou People’s Hospital, Affiliated Hospital of North Sichuan Medical College, Dali Prefecture People’s Hospital, Dongguan People’s Hospital, Ganzhou People’s Hospital, Guangyuan Central Hospital, 302 Hospital of China Guizhou Aviation Industry Group, Guiping People’s Hospital, Yangluo Campus‑The Third People’s Hospital of Hubei Province, Hubei Provincial Hospital of Traditional Chinese Medicine, Huainan First People’s Hospital, The First Hospital of Jilin University, Jiangmen Central Hospital, Jingdezhen First People’s Hospital, Liangshan Prefecture First People’s Hospital, Maoming Traditional Chinese Medicine Hospital, Meishan First People’s Hospital, Mianyang Central Hospital, Mianyang 404 Hospital, Nanbu County People’s Hospital, Nanjiang County People’s Hospital, Pingchang County People’s Hospital, Qianxinan Prefecture People’s Hospital, Qujing First People’s Hospital, Renshou County People’s Hospital, Shantou Central Hospital, Suining First People’s Hospital, Weihai Municipal Hospital, Wuhan No.1 Hospital, Affiliated Hospital of Southwest Medical University, Xiangtan Central Hospital, Xiangyang First People’s Hospital, Yibin First People’s Hospital, Yijishan Hospital of Wannan Medical College, The First People’s Hospital of Yunnan Province.

**Table S1. Predictive performance of ASPECTS, Tan, ASPECTS+Tan and AS-TCIS score for outcomes**

| **Outcome** | **C statistic (95% CI)** | **AIC** | **BIC** |
| --- | --- | --- | --- |
| **mRS 0-3** |  |  |  |
| ASPECTS | 0.73 (0.67–0.79) | 347.65 | 387.83 |
| Tan | 0.78 (0.72–0.83) | 326.15 | 366.33 |
| ASPECTS+Tan | 0.78 (0.72–0.83) | 325.85 | 369.68 |
| AS-TCIS | 0.8 (0.75–0.86) | 310.37 | 350.55 |
| **mRS 0-2** |  |  |  |
| ASPECTS | 0.69 (0.61–0.76) | 296.69 | 336.87 |
| Tan | 0.75 (0.69–0.81) | 277.92 | 318.1 |
| ASPECTS+Tan | 0.75 (0.69–0.81) | 279.19 | 323.02 |
| AS-TCIS | 0.77 (0.7–0.83) | 272.52 | 312.69 |
| **sICH** |  |  |  |
| ASPECTS | 0.71 (0.63–0.79) | 239.1 | 279.28 |
| Tan | 0.7 (0.62–0.79) | 238.59 | 278.76 |
| ASPECTS+Tan | 0.71 (0.63–0.8) | 239.96 | 283.79 |
| AS-TCIS | 0.71 (0.63–0.79) | 237.93 | 278.11 |
| **Mortality** |  |  |  |
| ASPECTS | 0.73 (0.67–0.79) | 366.54 | 406.71 |
| Tan | 0.78 (0.73–0.83) | 335.72 | 375.9 |
| ASPECTS+Tan | 0.78 (0.73–0.84) | 337.17 | 381 |
| AS-TCIS | 0.79 (0.74–0.85) | 331.85 | 372.03 |

**Figure S1. Flowchart of patient selection.**

NCCT, noncontrast computed tomography; MRI, magnetic resonance imaging; CTA, computed tomography angiography; CTP, computed tomography perfusion.
